# Draft Genome Sequence of *Streptococcus caviae* Strain Cavy grass 6^T^, Isolated from Domesticated Guinea Pig Fecal Samples

**DOI:** 10.1128/genomeA.00080-17

**Published:** 2017-03-30

**Authors:** Susakul Palakawong Na Ayudthaya, Ian P. G. Marshall, Lars Schreiber, Caroline M. Plugge

**Affiliations:** a Laboratory of Microbiology, Wageningen University, Wageningen, The Netherlands; b Center for Geomicrobiology, Department of Bioscience, Aarhus University, Aarhus C, Denmark

## Abstract

*Streptococcus caviae* strain Cavy grass 6^T^, isolated from fecal samples of pet guinea pigs, can metabolize a range of plant mono- and disaccharides, as well as polymeric carbohydrates. Here, we report the draft genome sequence of this strain, which comprises 2.11 Mb.

Guinea pigs (*Cavia porcellus*) are rodent animals of the family *Caviidae* that are native to South America and have been domesticated. Guinea pigs are monogastric herbivores with grass as their natural diet (1). The fact that guinea pigs consume major quantities of plant polymers suggests that their microbiome encodes active cellulolytic enzymes. Cellulose can be degraded by microorganisms such as bacteria and fungi. Those microorganisms produce extracellular cellulolytic enzymes that can hydrolyze cellulose to cellobiose and/or glucose, which can be further metabolized (2).

The genus *Streptococcus* comprises a wide variety of species that are found to inhabit a wide range of environments, including the gastrointestinal tract of herbivores (3). *S. caviae* strain Cavy grass 6^T^ is a cellobiose-degrading and lactate-producing bacterium. Strain Cavy grass 6^T^ was isolated from a fecal sample of a pet guinea pig with dried grass as substrate (4). *S. caviae* is a versatile bacterium that performs heterolactic fermentation, producing lactate, formate, acetate, and ethanol from a range of plant mono- and disaccharides, as well as polymeric carbohydrates (4).

Genomic DNA of strain Cavy grass 6^T^ was extracted from a culture grown on glucose (4) using the MasterPure complete DNA and RNA purification kit (Epicenter, USA). A sequencing library was prepared using the Nextera XT kit (Illumina, USA). The genome was sequenced using the Illumina MiSeq platform with MiSeq reagent kit version 3, generating approximately 4,582,712 paired-end reads of 300 bp. The quality of the generated reads was evaluated using FastQC version 0.11.5 (http://www.bioinformatics.babraham.ac.uk/projects/fastqc). The reads were trimmed and the adapters were removed using Trimmomatic version 0.36 (5) with the following parameters: CROP:289, HEADCROP:19, SLIDINGWINDOW:4:20, and MINLEN:100. Reads coding for 16S rRNA genes were extracted using BBMap version 35.82 and analyzed using SILVAngs (6) to determine contamination of the sequence data. Trimmed reads were assembled using SPAdes version 3.6.1 (7) with the following parameters: -k 21, 33, 55, 77, 99, and 127 and -careful. Scaffolds with a coverage below 35× (approx. 5.6%) were removed from the assembly. The final assembly of the draft genome contains 29 scaffolds with approximately 620-fold coverage.

The total draft genome is 2,108,609 bp and has an average G+C content of 42.25% and an *N*_50_ value of 217,434 bp. Draft genome completeness (99.61% complete) and contamination (0.12% potential contamination) were estimated by CheckM version 1.0.7 (8) using the gene marker set for the *Streptococcus* genus. Prokka version 1.12-beta (9) identified 1,995 protein-coding sequences, including 361 hypothetical proteins with no functional annotation. The genome contains four genes encoding rRNA (including two 5S, one 16S, and one 23S rRNAs), one tmRNA gene, and 48 tRNAs genes. Using the RAST server (10), 329 genes were found related to the degradation of carbohydrates, including amylose, cellobiose, glucose, maltose, maltodextrin, trehalose, and glycogen. Four genes were identified to be involved in self-defense mechanisms (CRISPRs: Cas 1, 2, and Csn 1, 2 families).

## Accession number(s).

The *S. caviae* strain Cavy grass 6^T^ genome sequence has been deposited at DDBJ/EMBL/GenBank under the accession number MOWR00000000. The version described in this paper is the first version, MOWR01000000.
